# Supplementary material for: Smooth Interpolating Curves with Local Control and Monotone Alternating Curvature
Source: Comput Graph Forum. 2022 Oct 6;41(5):25–38. doi: 10.1111/cgf.14600 (PMC9827861; doi:10.1111/cgf.14600)
Supplement: Supplementary file 1 — Supplement Material [file CGF-41-25-s001.zip › Local-Smooth-Interpolating-MonoCurvature/extern/clothoids/submodules/Utils/ThirdParties/fmt/fmt-8.0.1.zip!/fmt-8.0.1/doc/html/api.html]

API Reference — fmt 8.0.1 documentation


Toggle navigation

{fmt}

- 8.0.1
  - 8.0.1
  - 8.0.0
  - 7.1.3
- Contents
- Usage
- API
  (current)
- Syntax

# API Reference¶

The {fmt} library API consists of the following parts:

- fmt/core.h: the core API providing main formatting functions
  for `char`/UTF-8 with compile-time checks and minimal dependencies
- fmt/format.h: the full format API providing additional
  formatting functions and locale support
- fmt/ranges.h: formatting of ranges and tuples
- fmt/chrono.h: date and time formatting
- fmt/compile.h: format string compilation
- fmt/color.h: terminal color and text style
- fmt/os.h: system APIs
- fmt/ostream.h: `std::ostream` support
- fmt/printf.h: `printf` formatting
- fmt/xchar.h: optional `wchar_t` support

All functions and types provided by the library reside in namespace `fmt` and
macros have prefix `FMT_`.

## Core API¶

`fmt/core.h` defines the core API which provides main formatting functions for
`char`/UTF-8 with compile-time checks. It has minimal include dependencies for
better compile times. This header is only beneficial when using {fmt} as a
library and not in the header-only mode.

The following functions use format string syntax
similar to that of Python’s str.format.
They take *fmt* and *args* as arguments.

*fmt* is a format string that contains literal text and replacement
fields surrounded by braces `{}`. The fields are replaced with formatted
arguments in the resulting string. A function taking *fmt* doesn’t
participate in an overload resolution if the latter is not a string.

*args* is an argument list representing objects to be formatted.

template<typename ...`T`> auto `fmt::``format`(format\_string<T...> *fmt*, T&&... *args*) -> std::string¶
:   Formats `args` according to specifications in `fmt` and returns the result
    as a string.

    **Example**:

    ```
    #include <fmt/core.h>
    std::string message = fmt::format("The answer is {}", 42);
    ```

auto `fmt::``vformat`(string\_view *fmt*, format\_args *args*) -> std::string¶

template<typename `OutputIt`, typename ...`T`> auto `fmt::``format_to`(OutputIt *out*, format\_string<T...> *fmt*, T&&... *args*) -> OutputIt¶
:   Formats `args` according to specifications in `fmt`, writes the result to
    the output iterator `out` and returns the iterator past the end of the output
    range.

    **Example**:

    ```
    auto out = std::vector<char>();
    fmt::format_to(std::back_inserter(out), "{}", 42);
    ```

template<typename `OutputIt`, typename ...`T`> auto `fmt::``format_to_n`(OutputIt *out*, size\_t *n*, format\_string<T...> *fmt*, *const* T&... *args*) -> format\_to\_n\_result<OutputIt>¶
:   Formats `args` according to specifications in `fmt`, writes up to `n`
    characters of the result to the output iterator `out` and returns the total
    (not truncated) output size and the iterator past the end of the output range.

template<typename ...`T`> auto `fmt::``formatted_size`(format\_string<T...> *fmt*, T&&... *args*) -> size\_t¶
:   Returns the number of chars in the output of `format(fmt, args...)`.

template<typename `OutputIt`> *struct* `fmt::``format_to_n_result`¶
:   Public Members

    OutputIt `out`¶
    :   Iterator past the end of the output range.

    size\_t `size`¶
    :   Total (not truncated) output size.

template<typename ...`T`> void `fmt::``print`(format\_string<T...> *fmt*, T&&... *args*)¶
:   Formats `args` according to specifications in `fmt` and writes the output
    to `stdout`.

    **Example**:

    ```
    fmt::print("Elapsed time: {0:.2f} seconds", 1.23);
    ```

void `fmt::buffered_file::``vprint`(string\_view *format\_str*, format\_args *args*)¶

template<typename ...`T`> void `fmt::``print`(std::FILE \**f*, format\_string<T...> *fmt*, T&&... *args*)¶
:   Formats `args` according to specifications in `fmt` and writes the
    output to the file `f`.

    **Example**:

    ```
    fmt::print(stderr, "Don't {}!", "panic");
    ```

void `fmt::``vprint`(std::FILE \**f*, string\_view *fmt*, format\_args *args*)¶

### Compile-time Format String Checks¶

Compile-time checks are enabled when using `FMT_STRING`. They support built-in
and string types as well as user-defined types with `constexpr` `parse`
functions in their `formatter` specializations.

`FMT_STRING`(*s*)¶
:   Constructs a compile-time format string from a string literal *s*.

    **Example**:

    ```
    // A compile-time error because 'd' is an invalid specifier for strings.
    std::string s = fmt::format(FMT_STRING("{:d}"), "foo");
    ```

To force the use of compile-time checks, define the preprocessor variable
`FMT_ENFORCE_COMPILE_STRING`. When set, functions accepting `FMT_STRING`
will fail to compile with regular strings. Runtime-checked
formatting is still possible using `fmt::vformat`, `fmt::vprint`, etc.

### Named Arguments¶

template<typename `Char`, typename `T`> auto `fmt::``arg`(*const* Char \**name*, *const* T &*arg*) -> detail::named\_arg<Char, T>¶
:   Returns a named argument to be used in a formatting function.
    It should only be used in a call to a formatting function or
    `dynamic_format_arg_store::push_back`.

    **Example**:

    ```
    fmt::print("Elapsed time: {s:.2f} seconds", fmt::arg("s", 1.23));
    ```

Named arguments are not supported in compile-time checks at the moment.

### Argument Lists¶

You can create your own formatting function with compile-time checks and small
binary footprint, for example (https://godbolt.org/z/oba4Mc):

```
#include <fmt/format.h>

void vlog(const char* file, int line, fmt::string_view format,
          fmt::format_args args) {
  fmt::print("{}: {}: ", file, line);
  fmt::vprint(format, args);
}

template <typename S, typename... Args>
void log(const char* file, int line, const S& format, Args&&... args) {
  vlog(file, line, format,
      fmt::make_args_checked<Args...>(format, args...));
}

#define MY_LOG(format, ...) \
  log(__FILE__, __LINE__, FMT_STRING(format), __VA_ARGS__)

MY_LOG("invalid squishiness: {}", 42);
```

Note that `vlog` is not parameterized on argument types which improves compile
times and reduces binary code size compared to a fully parameterized version.

template<typename ...`Args`, typename `S`, typename `Char` = char\_t<S>> auto `fmt::``make_args_checked`(*const* S &*fmt*, *const* remove\_reference\_t<Args>&... *args*) -> format\_arg\_store<buffer\_context<Char>, remove\_reference\_t<Args>...>¶
:   Constructs a `format_arg_store` object that contains references
    to arguments and can be implicitly converted to `format_args`.
    If `fmt` is a compile-time string then `make_args_checked()` checks
    its validity at compile time.

template<typename `Context` = format\_context, typename ...`Args`> *constexpr* auto `fmt::``make_format_args`(*const* Args&... *args*) -> format\_arg\_store<Context, Args...>¶
:   Constructs a `format_arg_store` object that contains references to
    arguments and can be implicitly converted to `format_args`. `Context`
    can be omitted in which case it defaults to `context`.
    See `arg()` for lifetime considerations.

template<typename `Context`, typename ...`Args`> *class* `format_arg_store`¶
:   An array of references to arguments. It can be implicitly converted into
    `basic_format_args` for passing into type-erased formatting functions
    such as `vformat()`.

template<typename `Context`> *class* `dynamic_format_arg_store`¶

template<typename `Context`> *class* `fmt::``basic_format_args`¶
:   A view of a collection of formatting arguments. To avoid lifetime issues it
    should only be used as a parameter type in type-erased functions such as
    `vformat`:

    ```
    void vlog(string_view format_str, format_args args);  // OK
    format_args args = make_format_args(42);  // Error: dangling reference
    ```

    Public Functions

    template<typename ...`Args`> *constexpr* `basic_format_args`(*const* format\_arg\_store<Context, Args...> &*store*)¶
    :   Constructs a `basic_format_args()` object from `format_arg_store`.

    *constexpr* `basic_format_args`(*const* dynamic\_format\_arg\_store<Context> &*store*)¶
    :   Constructs a `basic_format_args()` object from
        `dynamic_format_arg_store`.

    *constexpr* `basic_format_args`(*const* format\_arg \**args*, int *count*)¶
    :   Constructs a `basic_format_args()` object from a dynamic set of arguments.

    auto `get`(int *id*) *const* -> format\_arg¶
    :   Returns the argument with the specified id.

*using* `fmt::``format_args` = basic\_format\_args<format\_context>¶
:   An alias to `basic_format_args<format_context>`.

template<typename `Context`> *class* `basic_format_arg`¶

template<typename `OutputIt`, typename `Char`> *class* `fmt::``basic_format_context`¶
:   Public Types

    *using* `char_type` = Char¶
    :   The character type for the output.

    Public Functions

    *constexpr* `basic_format_context`(OutputIt *out*, basic\_format\_args<basic\_format\_context> *ctx\_args*, detail::locale\_ref *loc* = detail::locale\_ref())¶
    :   Constructs a `basic_format_context` object.

        References to the arguments are stored in the object so make sure they have appropriate lifetimes.

*using* `fmt::``format_context` = buffer\_context<char>¶

### Compatibility¶

template<typename `Char`> *class* `fmt::``basic_string_view`¶
:   An implementation of `std::basic_string_view` for pre-C++17.

    It provides a subset of the API. `fmt::basic_string_view` is used for format strings even if `std::string_view` is available to prevent issues when a library is compiled with a different `-std` option than the client code (which is not recommended).

    Public Functions

    *constexpr* `basic_string_view`(*const* Char \**s*, size\_t *count*)¶
    :   Constructs a string reference object from a C string and a size.

    `basic_string_view`(*const* Char \**s*)¶
    :   Constructs a string reference object from a C string computing
        the size with `std::char_traits<Char>::length`.

    template<typename `Traits`, typename `Alloc`> `basic_string_view`(*const* std::basic\_string<Char, Traits, Alloc> &*s*)¶
    :   Constructs a string reference from a `std::basic_string` object.

    *constexpr* auto `data`() *const* -> *const* Char\*¶
    :   Returns a pointer to the string data.

    *constexpr* auto `size`() *const* -> size\_t¶
    :   Returns the string size.

*using* `fmt::``string_view` = basic\_string\_view<char>¶

### Locale¶

All formatting is locale-independent by default. Use the `'L'` format
specifier to insert the appropriate number separator characters from the
locale:

```
#include <fmt/core.h>
#include <locale>

std::locale::global(std::locale("en_US.UTF-8"));
auto s = fmt::format("{:L}", 1000000);  // s == "1,000,000"
```

## Format API¶

`fmt/format.h` defines the full format API providing additional formatting
functions and locale support.

### Formatting User-defined Types¶

To make a user-defined type formattable, specialize the `formatter<T>` struct
template and implement `parse` and `format` methods:

```
#include <fmt/format.h>

struct point { double x, y; };

template <> struct fmt::formatter<point> {
  // Presentation format: 'f' - fixed, 'e' - exponential.
  char presentation = 'f';

  // Parses format specifications of the form ['f' | 'e'].
  constexpr auto parse(format_parse_context& ctx) -> decltype(ctx.begin()) {
    // [ctx.begin(), ctx.end()) is a character range that contains a part of
    // the format string starting from the format specifications to be parsed,
    // e.g. in
    //
    //   fmt::format("{:f} - point of interest", point{1, 2});
    //
    // the range will contain "f} - point of interest". The formatter should
    // parse specifiers until '}' or the end of the range. In this example
    // the formatter should parse the 'f' specifier and return an iterator
    // pointing to '}'.

    // Parse the presentation format and store it in the formatter:
    auto it = ctx.begin(), end = ctx.end();
    if (it != end && (*it == 'f' || *it == 'e')) presentation = *it++;

    // Check if reached the end of the range:
    if (it != end && *it != '}')
      throw format_error("invalid format");

    // Return an iterator past the end of the parsed range:
    return it;
  }

  // Formats the point p using the parsed format specification (presentation)
  // stored in this formatter.
  template <typename FormatContext>
  auto format(const point& p, FormatContext& ctx) -> decltype(ctx.out()) {
    // ctx.out() is an output iterator to write to.
    return format_to(
        ctx.out(),
        presentation == 'f' ? "({:.1f}, {:.1f})" : "({:.1e}, {:.1e})",
        p.x, p.y);
  }
};
```

Then you can pass objects of type `point` to any formatting function:

```
point p = {1, 2};
std::string s = fmt::format("{:f}", p);
// s == "(1.0, 2.0)"
```

You can also reuse existing formatters via inheritance or composition, for
example:

```
enum class color {red, green, blue};

template <> struct fmt::formatter<color>: formatter<string_view> {
  // parse is inherited from formatter<string_view>.
  template <typename FormatContext>
  auto format(color c, FormatContext& ctx) {
    string_view name = "unknown";
    switch (c) {
    case color::red:   name = "red"; break;
    case color::green: name = "green"; break;
    case color::blue:  name = "blue"; break;
    }
    return formatter<string_view>::format(name, ctx);
  }
};
```

Since `parse` is inherited from `formatter<string_view>` it will recognize
all string format specifications, for example

```
fmt::format("{:>10}", color::blue)
```

will return `"      blue"`.

You can also write a formatter for a hierarchy of classes:

```
#include <type_traits>
#include <fmt/format.h>

struct A {
  virtual ~A() {}
  virtual std::string name() const { return "A"; }
};

struct B : A {
  virtual std::string name() const { return "B"; }
};

template <typename T>
struct fmt::formatter<T, std::enable_if_t<std::is_base_of<A, T>::value, char>> :
    fmt::formatter<std::string> {
  template <typename FormatCtx>
  auto format(const A& a, FormatCtx& ctx) {
    return fmt::formatter<std::string>::format(a.name(), ctx);
  }
};

int main() {
  B b;
  A& a = b;
  fmt::print("{}", a); // prints "B"
}
```

If a type provides both a `formatter` specialization and an implicit
conversion to a formattable type, the specialization takes precedence over the
conversion.

template<typename `Char`, typename `ErrorHandler` = detail::error\_handler> *class* `fmt::``basic_format_parse_context` : *private* fmt::detail::error\_handler¶
:   Parsing context consisting of a format string range being parsed and an
    argument counter for automatic indexing.
    You can use the `format_parse_context` type alias for `char` instead.

    Subclassed by fmt::detail::compile\_parse\_context< Char, ErrorHandler >, fmt::basic\_printf\_parse\_context< Char >

    Public Functions

    *constexpr* auto `begin`() *const* -> iterator¶
    :   Returns an iterator to the beginning of the format string range being parsed.

    *constexpr* auto `end`() *const* -> iterator¶
    :   Returns an iterator past the end of the format string range being parsed.

    void `advance_to`(iterator *it*)¶
    :   Advances the begin iterator to `it`.

    auto `next_arg_id`() -> int¶
    :   Reports an error if using the manual argument indexing; otherwise returns the next argument index and switches to the automatic indexing.

    void `check_arg_id`(int)¶
    :   Reports an error if using the automatic argument indexing; otherwise switches to the manual indexing.

### Literal-based API¶

The following user-defined literals are defined in `fmt/format.h`.

*constexpr* auto `fmt::``operator""_format`(*const* char \**s*, size\_t *n*) -> detail::udl\_formatter<char>¶
:   User-defined literal equivalent of `fmt::format()`.

    **Example**:

    ```
    using namespace fmt::literals;
    std::string message = "The answer is {}"_format(42);
    ```

*constexpr* auto `fmt::``operator""_a`(*const* char \**s*, size\_t) -> detail::udl\_arg<char>¶
:   User-defined literal equivalent of `fmt::arg()`.

    **Example**:

    ```
    using namespace fmt::literals;
    fmt::print("Elapsed time: {s:.2f} seconds", "s"_a=1.23);
    ```

### Utilities¶

template<typename `T`> auto `fmt::``ptr`(T *p*) -> *const* void\*¶
:   Converts `p` to `const void*` for pointer formatting.

    **Example**:

    ```
    auto s = fmt::format("{}", fmt::ptr(p));
    ```

template<typename `T`> auto `fmt::``ptr`(*const* std::unique\_ptr<T> &*p*) -> *const* void\*¶

template<typename `T`> auto `fmt::``ptr`(*const* std::shared\_ptr<T> &*p*) -> *const* void\*¶

template<typename `T`> auto `fmt::``to_string`(*const* T &*value*) -> std::string¶
:   Converts *value* to `std::string` using the default format for type *T*.

    **Example**:

    ```
    #include <fmt/format.h>

    std::string answer = fmt::to_string(42);
    ```

template<typename `Char`> auto `fmt::``to_string_view`(*const* Char \**s*) -> basic\_string\_view<Char>¶

template<typename `Range`> auto `fmt::``join`(Range &&*range*, string\_view *sep*) -> join\_view<detail::iterator\_t<Range>, detail::sentinel\_t<Range>>¶
:   Returns an object that formats `range` with elements separated by `sep`.

    **Example**:

    ```
    std::vector<int> v = {1, 2, 3};
    fmt::print("{}", fmt::join(v, ", "));
    // Output: "1, 2, 3"
    ```

    `fmt::join` applies passed format specifiers to the range elements:

    ```
    fmt::print("{:02}", fmt::join(v, ", "));
    // Output: "01, 02, 03"
    ```

template<typename `It`, typename `Sentinel`> auto `fmt::``join`(It *begin*, Sentinel *end*, string\_view *sep*) -> join\_view<It, Sentinel>¶
:   Returns an object that formats the iterator range `[begin, end)` with elements separated by `sep`.

template<typename `T`> *class* `fmt::detail::``buffer`¶
:   A contiguous memory buffer with an optional growing ability. It is an internal
    class and shouldn’t be used directly, only via `basic_memory_buffer`.

    Subclassed by fmt::basic\_memory\_buffer< wchar\_t >, fmt::basic\_memory\_buffer< T, SIZE, Allocator >, fmt::detail::iterator\_buffer< OutputIt, T, Traits >, fmt::detail::iterator\_buffer< T \*, T >

    Public Functions

    auto `size`() *const* -> size\_t¶
    :   Returns the size of this buffer.

    auto `capacity`() *const* -> size\_t¶
    :   Returns the capacity of this buffer.

    auto `data`() -> T\*¶
    :   Returns a pointer to the buffer data.

    auto `data`() *const* -> *const* T\*¶
    :   Returns a pointer to the buffer data.

    void `clear`()¶
    :   Clears this buffer.

    template<typename `U`> void `append`(*const* U \**begin*, *const* U \**end*)¶
    :   Appends data to the end of the buffer.

template<typename `T`, size\_t `SIZE` = inline\_buffer\_size, typename `Allocator` = std::allocator<T>> *class* `fmt::``basic_memory_buffer` : *public* fmt::detail::buffer<T>¶
:   A dynamically growing memory buffer for trivially copyable/constructible types
    with the first `SIZE` elements stored in the object itself.

    You can use the `memory_buffer` type alias for `char` instead.

    **Example**:

    ```
    fmt::memory_buffer out;
    format_to(out, "The answer is {}.", 42);
    ```

    This will append the following output to the `out` object:

    ```
    The answer is 42.
    ```

    The output can be converted to an `std::string` with `to_string(out)`.

    Public Functions

    `basic_memory_buffer`(basic\_memory\_buffer &&*other*)¶
    :   Constructs a `fmt::basic_memory_buffer` object moving the content
        of the other object to it.

    auto `operator=`(basic\_memory\_buffer &&*other*) -> basic\_memory\_buffer&¶
    :   Moves the content of the other `basic_memory_buffer` object to this one.

    void `resize`(size\_t *count*)¶
    :   Resizes the buffer to contain *count* elements.

        If T is a POD type new elements may not be initialized.

    void `reserve`(size\_t *new\_capacity*)¶
    :   Increases the buffer capacity to *new\_capacity*.

    Protected Functions

    void `grow`(size\_t *size*) *final*¶
    :   Increases the buffer capacity to hold at least *capacity* elements.

### System Errors¶

{fmt} does not use `errno` to communicate errors to the user, but it may call
system functions which set `errno`. Users should not make any assumptions
about the value of `errno` being preserved by library functions.

template<typename ...`T`> auto `fmt::``system_error`(int *error\_code*, format\_string<T...> *fmt*, T&&... *args*) -> std::system\_error¶
:   Constructs `std::system_error` with a message formatted with
    `fmt::format(fmt, args...)`.

    > *error\_code* is a system error code as given by `errno`.

    **Example**:

    ```
    // This throws std::system_error with the description
    //   cannot open file 'madeup': No such file or directory
    // or similar (system message may vary).
    const char* filename = "madeup";
    std::FILE* file = std::fopen(filename, "r");
    if (!file)
      throw fmt::system_error(errno, "cannot open file '{}'", filename);
    ```

void `fmt::``format_system_error`(detail::buffer<char> &*out*, int *error\_code*, *const* char \**message*)¶
:   Formats an error message for an error returned by an operating system or a
    language runtime, for example a file opening error, and writes it to *out*.
    The format is the same as the one used by `std::system_error(ec, message)`
    where `ec` is `std::error_code(error_code, std::generic_category()})`.
    It is implementation-defined but normally looks like:

    ```
    <message>: <system-message>
    ```

    where *<message>* is the passed message and *<system-message>* is the system
    message corresponding to the error code.
    *error\_code* is a system error code as given by `errno`.

### Custom Allocators¶

The {fmt} library supports custom dynamic memory allocators.
A custom allocator class can be specified as a template argument to
`fmt::basic_memory_buffer`:

```
using custom_memory_buffer =
  fmt::basic_memory_buffer<char, fmt::inline_buffer_size, custom_allocator>;
```

It is also possible to write a formatting function that uses a custom
allocator:

```
using custom_string =
  std::basic_string<char, std::char_traits<char>, custom_allocator>;

custom_string vformat(custom_allocator alloc, fmt::string_view format_str,
                      fmt::format_args args) {
  custom_memory_buffer buf(alloc);
  fmt::vformat_to(buf, format_str, args);
  return custom_string(buf.data(), buf.size(), alloc);
}

template <typename ...Args>
inline custom_string format(custom_allocator alloc,
                            fmt::string_view format_str,
                            const Args& ... args) {
  return vformat(alloc, format_str, fmt::make_format_args(args...));
}
```

The allocator will be used for the output container only. Formatting functions
normally don’t do any allocations for built-in and string types except for
non-default floating-point formatting that occasionally falls back on
`sprintf`.

## Ranges and Tuple Formatting¶

The library also supports convenient formatting of ranges and tuples:

```
#include <fmt/ranges.h>

std::tuple<char, int, float> t{'a', 1, 2.0f};
// Prints "('a', 1, 2.0)"
fmt::print("{}", t);
```

NOTE: currently, the overload of `fmt::join` for iterables exists in the main
`format.h` header, but expect this to change in the future.

Using `fmt::join`, you can separate tuple elements with a custom separator:

```
#include <fmt/ranges.h>

std::tuple<int, char> t = {1, 'a'};
// Prints "1, a"
fmt::print("{}", fmt::join(t, ", "));
```

## Date and Time Formatting¶

`fmt/chrono.h` provides formatters for

- std::chrono::duration
- std::chrono::time\_point
- std::tm

The format syntax is described in Chrono Format Specifications.

**Example**:

```
#include <fmt/chrono.h>

int main() {
  std::time_t t = std::time(nullptr);

  // Prints "The date is 2020-11-07." (with the current date):
  fmt::print("The date is {:%Y-%m-%d}.", fmt::localtime(t));

  using namespace std::literals::chrono_literals;

  // Prints "Default format: 42s 100ms":
  fmt::print("Default format: {} {}\n", 42s, 100ms);

  // Prints "strftime-like format: 03:15:30":
  fmt::print("strftime-like format: {:%H:%M:%S}\n", 3h + 15min + 30s);
}
```

std::tm `fmt::``localtime`(std::time\_t *time*)¶
:   Converts given time since epoch as `std::time_t` value into calendar time, expressed in local time.

    Unlike `std::localtime`, this function is thread-safe on most platforms.

std::tm `fmt::``gmtime`(std::time\_t *time*)¶
:   Converts given time since epoch as `std::time_t` value into calendar time, expressed in Coordinated Universal Time (UTC).

    Unlike `std::gmtime`, this function is thread-safe on most platforms.

## Format string compilation¶

`fmt/compile.h` provides format string compilation support when using
`FMT_COMPILE`. Format strings are parsed, checked and converted into efficient
formatting code at compile-time. This supports arguments of built-in and string
types as well as user-defined types with `constexpr` `parse` functions in
their `formatter` specializations. Format string compilation can generate more
binary code compared to the default API and is only recommended in places where
formatting is a performance bottleneck.

`FMT_COMPILE`(*s*)¶
:   Converts a string literal *s* into a format string that will be parsed at
    compile time and converted into efficient formatting code. Requires C++17
    `constexpr if` compiler support.

    **Example**:

    ```
    // Converts 42 into std::string using the most efficient method and no
    // runtime format string processing.
    std::string s = fmt::format(FMT_COMPILE("{}"), 42);
    ```

## Terminal color and text style¶

`fmt/color.h` provides support for terminal color and text style output.

template<typename `S`, typename ...`Args`> void `fmt::``print`(*const* text\_style &*ts*, *const* S &*format\_str*, *const* Args&... *args*)¶
:   Formats a string and prints it to stdout using ANSI escape sequences to
    specify text formatting.

    **Example**:

    ```
    fmt::print(fmt::emphasis::bold | fg(fmt::color::red),
               "Elapsed time: {0:.2f} seconds", 1.23);
    ```

text\_style `fmt::``fg`(detail::color\_type *foreground*)¶
:   Creates a text style from the foreground (text) color.

text\_style `fmt::``bg`(detail::color\_type *background*)¶
:   Creates a text style from the background color.

## System APIs¶

*class* `fmt::``ostream` : *private* fmt::detail::buffer<char>¶
:   A fast output stream which is not thread-safe.

    Public Functions

    template<typename ...`T`> void `print`(format\_string<T...> *fmt*, T&&... *args*)¶
    :   Formats `args` according to specifications in `fmt` and writes the output to the file.

    Friends

    template<typename ...`T`> ostream `output_file`(cstring\_view *path*, T... *params*)¶
    :   Opens a file for writing. Supported parameters passed in *params*:

        - `<integer>`: Flags passed to open
          (`file::WRONLY | file::CREATE` by default)
        - `buffer_size=<integer>`: Output buffer size

        **Example**:

        ```
        auto out = fmt::output_file("guide.txt");
        out.print("Don't {}", "Panic");
        ```

## `std::ostream` Support¶

`fmt/ostream.h` provides `std::ostream` support including formatting of
user-defined types that have an overloaded insertion operator (`operator<<`):

```
#include <fmt/ostream.h>

class date {
  int year_, month_, day_;
public:
  date(int year, int month, int day): year_(year), month_(month), day_(day) {}

  friend std::ostream& operator<<(std::ostream& os, const date& d) {
    return os << d.year_ << '-' << d.month_ << '-' << d.day_;
  }
};

std::string s = fmt::format("The date is {}", date(2012, 12, 9));
// s == "The date is 2012-12-9"
```

{fmt} only supports insertion operators that are defined in the same namespaces
as the types they format and can be found with the argument-dependent lookup.

template<typename `S`, typename ...`Args`, typename `Char` = enable\_if\_t<detail::is\_string<S>::value, char\_t<S>>> void `fmt::``print`(std::basic\_ostream<Char> &*os*, *const* S &*format\_str*, Args&&... *args*)¶
:   Prints formatted data to the stream *os*.

    **Example**:

    ```
    fmt::print(cerr, "Don't {}!", "panic");
    ```

## `printf` Formatting¶

The header `fmt/printf.h` provides `printf`-like formatting functionality.
The following functions use printf format string syntax with
the POSIX extension for positional arguments. Unlike their standard
counterparts, the `fmt` functions are type-safe and throw an exception if an
argument type doesn’t match its format specification.

template<typename `S`, typename ...`T`> auto `fmt::``printf`(*const* S &*fmt*, *const* T&... *args*) -> int¶
:   Prints formatted data to `stdout`.

    **Example**:

    ```
    fmt::printf("Elapsed time: %.2f seconds", 1.23);
    ```

template<typename `S`, typename ...`T`, typename `Char` = char\_t<S>> auto `fmt::``fprintf`(std::FILE \**f*, *const* S &*fmt*, *const* T&... *args*) -> int¶
:   Prints formatted data to the file *f*.

    **Example**:

    ```
    fmt::fprintf(stderr, "Don't %s!", "panic");
    ```

template<typename `S`, typename ...`T`, typename `Char` = enable\_if\_t<detail::is\_string<S>::value, char\_t<S>>> auto `fmt::``sprintf`(*const* S &*fmt*, *const* T&... *args*) -> std::basic\_string<Char>¶
:   Formats arguments and returns the result as a string.

    **Example**:

    ```
    std::string message = fmt::sprintf("The answer is %d", 42);
    ```

## `wchar_t` Support¶

The optional header `fmt/wchar_t.h` provides support for `wchar_t` and
exotic character types.

template<typename `T`> *struct* `is_char` : *public* std::false\_type¶
:   Specifies if `T` is a character type.

    Can be specialized by users.

*using* `fmt::``wstring_view` = basic\_string\_view<wchar\_t>¶

*using* `fmt::``wformat_context` = buffer\_context<wchar\_t>¶

template<typename `T`> auto `fmt::``to_wstring`(*const* T &*value*) -> std::wstring¶
:   Converts *value* to `std::wstring` using the default format for type *T*.

## Compatibility with C++20 `std::format`¶

{fmt} implements nearly all of the C++20 formatting library with the following
differences:

- Names are defined in the `fmt` namespace instead of `std` to avoid
  collisions with standard library implementations.
- Width calculation doesn’t use grapheme clusterization. The latter has been
  implemented in a separate branch but hasn’t been integrated yet.
- Most C++20 chrono types are not supported yet.

© Copyright 2012-present, Victor Zverovich.
Created using Sphinx 3.3.0.
